# Resveratrol-Mediated Repression and Reversion of Prostatic Myofibroblast Phenoconversion

**DOI:** 10.1371/journal.pone.0158357

**Published:** 2016-07-01

**Authors:** Mehrnaz Gharaee-Kermani, Bethany B. Moore, Jill A. Macoska

**Affiliations:** 1 Department of Biology, Center for Personalized Cancer Therapy, The University of Massachusetts, Boston, 02125, United States of America; 2 Department of Internal Medicine, The University of Michigan, Ann Arbor, Michigan, 48109, United States of America; National Cancer Institute, UNITED STATES

## Abstract

**Background:**

Resveratrol, a phytoalexin found in berries, peanuts, grapes, and red wine, inhibits oxidation, inflammation, and cell proliferation and collagen synthesis in multiple cell types and or animal models. It represses collagen deposition in the vasculature, heart, lung, kidney, liver, and esophagus in animal models and may have some utility as an anti-fibrotic. Recent studies have shown that increased collagen deposition and tissue stiffness in the peri-urethral area of the prostate are associated with lower urinary tract dysfunction (LUTD) and urinary obstructive symptoms. The aim of this study was to determine whether Resveratrol might be useful to inhibit or revert TGF**β**- and/or CXCL12-mediated myofibroblast phenoconversion of prostate fibroblasts *in vitro*, and therefore whether the use of anti-fibrotic therapeutics might be efficacious for the treatment of LUTD.

**Methods:**

Primary prostate and lung tissues were explanted and fibroblast monolayers expanded *in vitro*. Primary and N1 immortalized prostate stromal fibroblasts, as well as primary fibroblasts cultured from a normal lung and one affected by idiopathic pulmonary fibrosis (IPF) for comparison, were grown in serum–free defined media supplemented with vehicle, TGF**β** or CXCL12, pre- or post-treatment with Resveratrol, and were evaluated using immunofluorescence for alpha smooth muscle actin (**α**SMA) and collagen I (COL1) protein expression and assessed for cell proliferation, apoptosis, and COL1 and EGR1 transcript expression.

**Results:**

This study showed that low concentrations of Resveratrol (≤50 μM) had no effect on N1 or primary prostate fibroblast cell proliferation, apoptosis, or COL1 or EGR1 gene transcription but repressed and reversed myofibroblast phenoconversion. As expected, these same effects were observed for IPF lung fibroblasts though higher levels of Resveratrol (≥100uM) were required. Taken together, these data suggest that, like lung fibroblasts, prostate fibroblast to myofibroblast phenoconversion can be both repressed and reversed by Resveratrol treatment. Thus, anti-fibrotic therapeutics might be efficacious for the treatment of LUTD.

## Introduction

Fibrosis is an aberrant version of the normal wound healing process characterized by myofibroblast accumulation, collagen deposition, extracellular matrix (ECM) remodeling and tissue rigidization [1–3]. Numerous studies show that aging- and inflammation-associated fibrotic changes in tissue architecture contribute to dysfunction and disease in multiple organ systems, including pancreatic dysfunction in type 2 diabetes [4, 5], idiopathic pulmonary fibrosis (IPF) [6, 7], cirrhotic nonalcoholic fatty acid liver disease [8, 9] and Crohn's disease, which is part of the spectrum disorder termed inflammatory bowel disease[10, 11]. Several cell types, including fibroblasts, pericytes, fibrocytes, and mesencymal cells, may be capable of differentiating into myofibroblasts[12]. The common hallmarks of myofibroblast differentiation are expression of **α**SMA and COL1, the latter of which is a large component of myofibroblast-secreted extracellular matrix (ECM).

Recent studies from our laboratory have showed that the peri-urethral area of the prostate is subject to myofibroblast accumulation and persistence which remodels the ECM, increases tissue rigidity/stiffness, and that these phenomena are associated with urinary voiding dysfunction [13, 14]. Current clinical management for urinary voiding dysfunction in men includes surgical tissue ablation and medical approaches using anti-androgens or smooth-muscle relaxers. However, surgical tissue ablation often provides only a temporary measure for relief as prostate tissue continues to proliferate and obstruct urethral function. Medical management, though effective for some patients, is ineffective or intolerable for others [15, 16]. Therefore, lower urinary tract fibrosis may comprise another but untreated contributing pathobiology to urinary voiding dysfunction.

The phytoalexin Resveratrol (C_14_H_12_O_3_) ameliorates myofibroblast phenoconversion of fibroblasts from several organ systems including the liver, pancreas, and lung [17–19]. The aim of the current study was to determine whether Resveratrol might affect the repression or reversal of TGF**β**- and/or CXCL12-promoted prostate myofibroblast phenoconversion in vitro, and whether these effects could be achieved with low or no toxicity to normal fibroblastic cells. The goal of the study was to assess whether anti-fibrotic therapeutics like Resveratrol might have efficacy for the treatment of lower urinary tract fibrosis as a contributing pathobiology to urinary voiding dysfunction.

## Materials and Methods

### Cell Culture

Lung fibroblasts were explanted and grown from lung tissues obtained from patients undergoing diagnostic surgical lung biopsy that were positive for diagnosis of IPF (IPF fibroblasts) or from patients undergoing thoracic surgery for non-fibrotic lung diseases (normal lung fibroblasts). Prostate tissue from transurethral resection of the prostate (TURP) for benign prostatic hyperplasia (BPH) treatment was received from the operating room within 4 hrs of resection in sterile 10% RPMI media and processed immediately for primary explant and culture as previously described [14]. Primary fibroblast cells were isolated and cultured as described previously, and were used at low passage number (<10 culture passages) [14]. N1 cells are immortalized, nontransformed prostate stromal fibroblasts that grow continuously in culture but do not form colonies in soft agar or tumors in immuno-compromised mice [20]. Primary and N1 immortalized prostate stromal fibroblasts were maintained in 5% HIE media Ham’s F12 or in defined serum- free (SF) HIE media as previously described All studies were performed using cultured fibroblasts between the fifth and tenth passages. Cells were maintained in Dulbecco’s modified Eagle medium (DMEM) with 20% fetal calf serum (FCS), penicillin, streptomycin, glutamine and fungizone as described previously [21] [20]. In all cases, written informed consent was obtained from all subjects for this study in compliance with the requirements of the University of Michigan Institutional Review Board, and the entire study was approved by the University of Michigan Institutional Review Board.

### Myofibroblast Phenoconversion

Primary IPF and normal lung fibroblasts, or primary and N1 prostate stromal fibroblasts were grown to 70% confluence, washed twice with Phosphate Buffered Saline (PBS), and then grown for 24 hours in DMEM supplemented with 0.5% FBS (lung fibroblast) or SF HIE media (prostate fibroblast). The cells were then washed with additional media and treated with 5 ng/ml TGF**β**1 (Cell Signaling, Beverly, MA) or vehicle (20 mM citrate pH 3.0), or 100 pM CXCL12 (R&D Systems, Minneapolis, MN), or vehicle (0.1 bovine serum albumin in PBS) for 4, 8, 12, 24 or 48 hrs as previously described [14].

### Resveratrol Treatment

A 100 mM stock solution was prepared by dissolving Resveratrol (Cat. No R5010, Sigma Chemical, St. Louis, MO) in sterile cell culture-grade DMSO (Sigma). Working dilutions were made in culture medium immediately before use, while maintaining a uniform 0.1% concentration of DMSO [22]. Control cultures were exposed to 0.1% DMSO in medium. Resveratrol and its stock solution were stored at 20°C, protected from light. To examine the potential for Resveratrol-mediated repression of myofibroblast phenoconversion, cells were pre-treated in SF-defined media with Resveratrol at 10, 20, 25, 30, 40, 50,75, 100, 125, 150, or 200 μM for 2 hrs followed by treatment with TGF**β** (4ng/ml) or CXCL12 (100 pM) for 2, 4, 8, 12, 24 and 48 hr. To examine the potential for Resveratrol-mediated reversion of myofibroblast phenoconversion, cells were transitioned to SF-media for 24 hrs, followed by stimulation TGF**β** (4ng/ml) or CXCL12 (100 pM) for 24 hrs followed by post-treatment with Resveratrol at 10, 20, 25, 30, 40, 50,75, 100, 125, 150, or 200 μM for 24 hrs.

### Dose-Response Assays

WST-1 assays were conducted as previously described [23]. Cells were grown in SF media supplemented with 1–200 nM Resveratrol for 24, 48 or 72 hours. The IC_50_ for Resveratrol was assessed at 50% cell survival.

### Proliferation Assays

WST-1assays were conducted as previously described [23]. Cells were grown in SF media supplemented with or without Resveratrol at 10–200 uM for 2 hrs followed by stimulation of TGF**β** (4ng/ml), or CXCL12 (100 pM/ml) for 24–48 hr. Proliferation was assessed at 24 and 48 hr by WST assay (Roche, USA, Cat. No. 11644807001). Average cell numbers and standard deviations were calculated. Significant differences between datasets was assessd at p≤0.05.

### Apoptosis Assays

Lung and prostate fibroblasts were seeded in 96-well plates at 10000 cells/well and treated with 10–200 μM Resveratrol with or without TGF**β** or CXCL12. At the end of incubation, the cells were centrifuged, and the cell pellet suspended in lysis buffer. After centrifugation, the supernatant was transferred into streptavidin-coated plates for apoptosis analysis using Cell-Death-Detection ELISA kit (Roche, USA, Cat. No. 11774425001).

### Immunofluorescence Assays

Cells were plated on chamber slides coated with 10 μg/ml fibronectin (Sigma-Aldrich, St. Louis, MO), then treated as above and subjected to immunofluorescence as previously described using FITC-conjugated mouse monoclonal anti-α-smooth muscle actin (**α**SMA) (Sigma-Aldrich, St. Louis, MO), and biotin conjugated rabbit polyclonal anti-collagen type 1 (COL1) (Rockland Immunochemicals, Gilbertsville, PA), PE-Cy 5 streptavidin (BD Pharmingen San Diego, CA) secondary antibodies, and control mouse IgG2a (Sigma-Aldrich, St. Louis, MO), and rabbit IgG biotin conjugate (Rockland Immunochemicals, Gilbertsville, PA) [14]. Photomicrographs were taken on an Olympus BX53 fluorescence microscope.

### RNA Extraction and Quantitative Real Time PCR (qRT-PCR)

Lung, N1 and primary prostate fibroblasts cells were treated as above and RNA extracted using Trizol reagent (Invitrogen, Carlsbad, CA). Purified RNA was treated with DNase and subjected to qRT-PCR as previously described using an Applied Biosystems QuantStudio instrument and reagents [14]. Reactions were performed in triplicate, including no template controls and amplification of an endogenous control transcript, Larger Ribosomal Protein (RPLPO) to assess template concentration, and the results averaged, statistically analyzed using t-tests, and graphed. Cycle numbers to threshold were calculated by subtracting averaged control from averaged experimental values. Collagen 1A1 (COL1), EGR1 and EGR2 transcript levels were normalized to those of RPLPO using the Pfaffl method [24]. Gene-specific Assays-on-Demand (Applied Biosystems) used were Hs0016400_m1 for COL1, Hs00152928_m1 for EGR1, Hs00166165_m1 for EGR2, and Hs99999902_m1 for RPLPO (Applied Biosystems, Carlsbad, CA).

### Statistical Analysis

Averages and standard deviations were calculated and compared using 2-tailed Student’s t-tests. In all tests, p ≤ .05 was considered statistically significant.

## Results

### Prostate Fibroblast Sensitivity to Resveratrol

**Fig 1:**
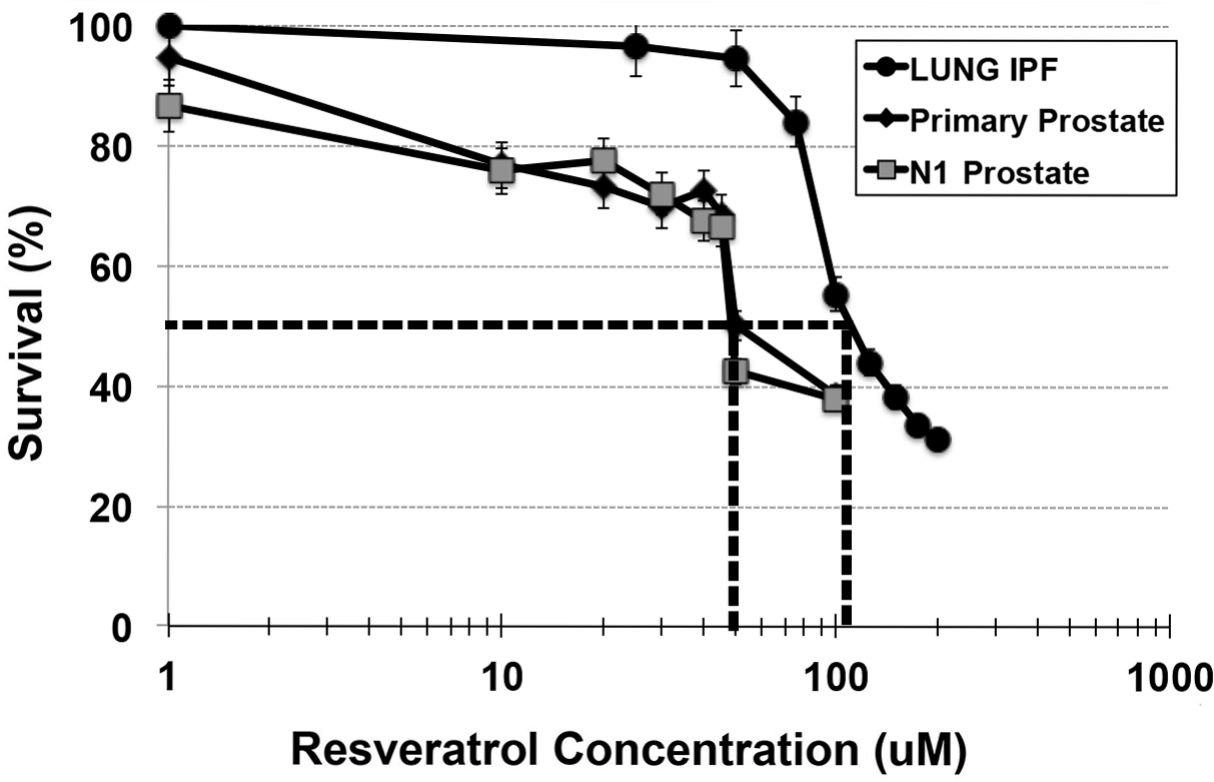
Establishment of Resveratrol IC50. N1 immortalized prostate (A), primary prostate (B) or Idiopathic Pulmonary Fibrosis (IPF) lung fibroblasts were treated with ascending concentrations of Resveratrol for 24 hr. Dotted lines are drawn from 50% cellular survival on Y-axis to corresponding Resveratrol concentration on X-axis. The IC50 for each cell line is the concentration of Resveratrol corresponding to 50% cellular survival; 50 uM for prostate fibroblasts, 100uM for IPF fibroblasts.

In order to determine Resveratrol toxicity for prostate fibroblasts, both N1 immortalized and primary prostate fibroblasts were assessed for %survival at doses of Resveratrol ranging from 1 to 100uM. As seen in Fig 1, survival dropped sharply to 50% for both N1 and primary cells at 50uM Resveratrol, and to ~20% at 100uM Resveratrol. Therefore, the apparent IC50 of Resveratrol for the prostate cells was 50uM. The IPF lung cells demonstrated somewhat more resiliency against Resveratrol, with an apparent IC50 of 100uM. Based on these data, further studies utilized 50uM Resveratrol for the prostate cells and 100uM for the IPF lung cells.

### High Doses of Resveratrol Repress Cellular Proliferation

**Fig 2:**
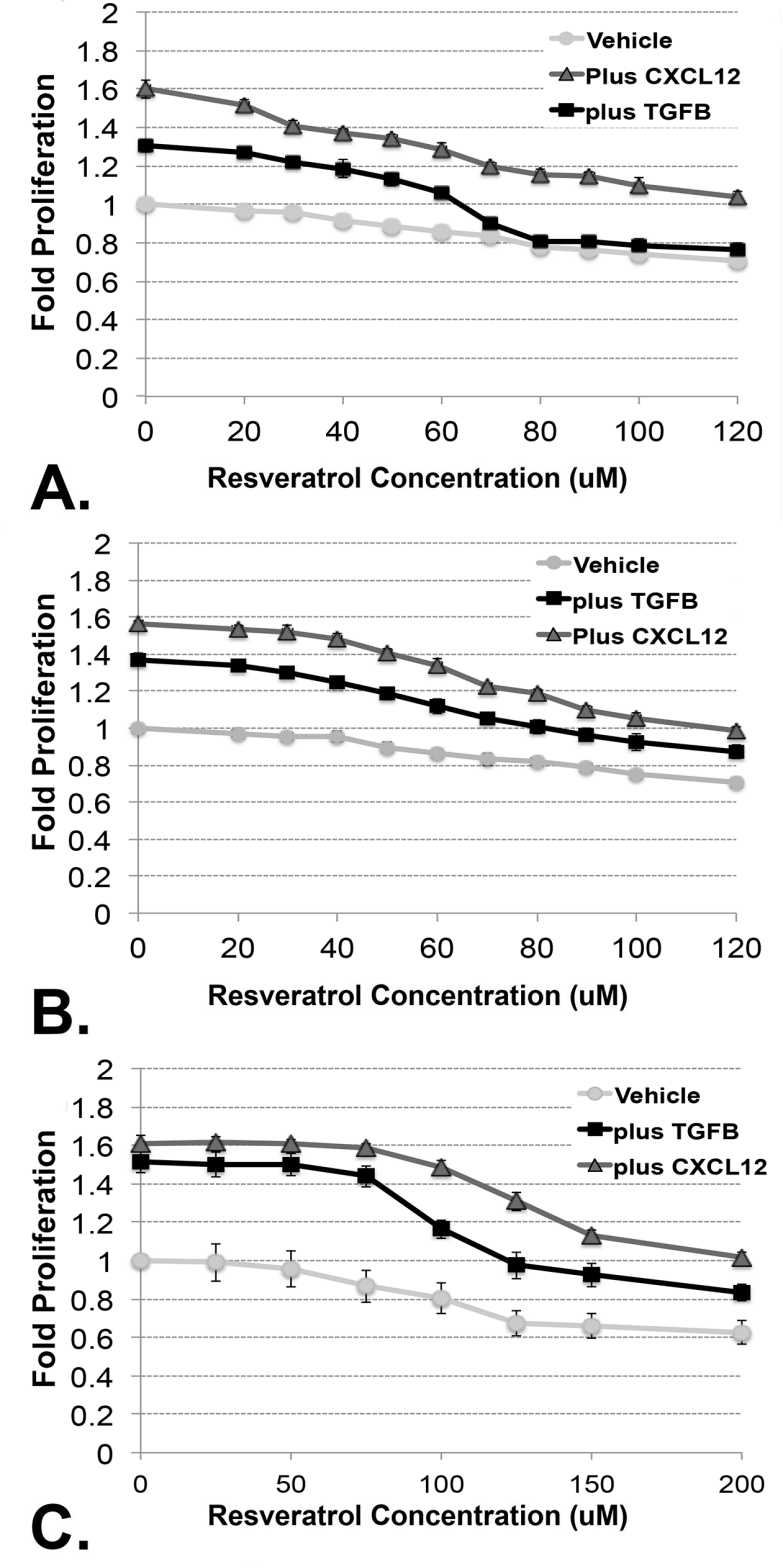
High Doses of Resveratrol Reduce Cellular Proliferation. N1 immortalized prostate (A), primary prostate (B) or Idiopathic Pulmonary Fibrosis (IPF) lung fibroblasts were cultured in SF media supplemented with vehicle or increasing doses of Resveratrol (10–200 μM) for 24 hours, then assessed using a WST assay. None of the cell lines tested proliferated in media supplemented with increasing doses of Resveratrol (light gray lines). N1 (A) and primary (B) prostate fibroblasts as well as IPF lung fibroblasts (C) proliferated in response to CXCL12 or TGF**β** though this response was ablated in media supplemented with increasing concentrations of Resveratrol. In all cases, CXCL12 promoted a more robust proliferative response than TGF**β**.

N1 and primary prostate fibroblasts and primary IPF lung fibroblasts were cultured in serum-free media supplemented with vehicle or increasing doses of Resveratrol (20–200 μM) for 24 hours. N1 immortalized and primary prostate cells failed to proliferate in media supplemented with increasing doses of Resveratrol (Fig 2A and 2B). In contrast, N1 immortalized and primary prostate cells proliferated when treated with 100pM CXCL12 but this effect was reduced in the presence of ≥50 μM Resveratrol and nearly ablated in the presence of 100 μM Resveratrol (Fig 2A and 2B). N1 immortalized and primary prostate cells also proliferated in response to treatment with 4ng/ml TGF**β** though to a lesser extent than that observed in response to 100pM CXCL12 (Fig 2D and 2E). IPF lung fibroblasts also failed to proliferate in media supplemented with Resveratrol. These cells demonstrated equivalent proliferative responses upon treatment with 100pM CXCL12 (Fig 2C) or 4 ng/ml TGF**β** (Fig 2D), though these responses were diminished in media supplemented with ≥100 μM Resveratrol. Taken together, these studies suggest that, even in the presence of growth factors, high doses of Resveratrol can exert an anti-proliferative effect.

### High Doses of Resveratrol Induce Apoptosis

**Fig 3:**
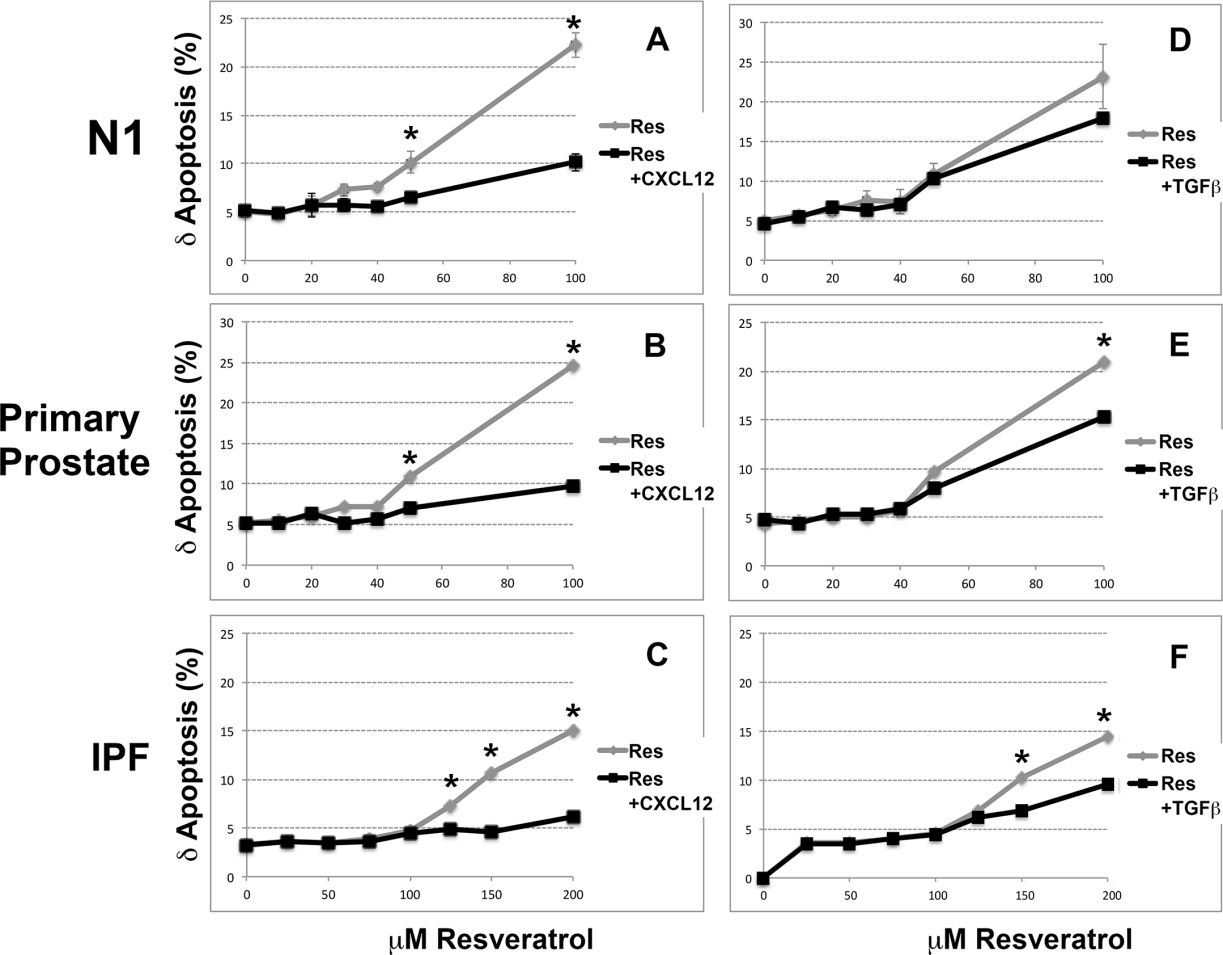
High Doses of Resveratrol Induce Apoptosis. N1 (**A, D**) and primary (**B, E**) prostate fibroblasts and primary IPF lung (**C, F**) fibroblasts were cultured in SF supplemented with vehicle or increasing doses of Resveratrol (10–200 μM) for 24 hours, then assayed for apoptosis using the Roche assay. Each point indicates the mean and SD of the data collected from triplets samples. All cells tested demonstrated increased apoptosis from a basal level of ~5% to 20–25% upon supplementation with ≥ 50 μM Resveratrol. However, co-treatment with CXCL12 or TGF**β** reduced the apoptotic rate for all cells tested by 5–10%.

Studies were next carried out to determine whether the observed reduction in cellular proliferation at high doses of Resveratrol reflected increased apoptosis. For these experiments, N1 and primary prostate fibroblasts and primary IPF lung fibroblasts were cultured in SF media supplemented with vehicle or increasing doses of Resveratrol (10–200 μM) for 24 hours, then assayed for apoptosis. All cells tested demonstrated increased apoptosis from a basal level of ~5% to 20–25% upon supplementation with ≥ 50 μM Resveratrol (Fig 3). Co-treatment with 100pM CXCL12 reduced the apoptotic rate for all cells tested by 5–10% (Fig 3A, 3B and 3C). Co-treatment with TGF**β** reduced the apoptotic rate by ~5% for all cells tested (Fig 3D, 3E and 3F). Moreover, whereas N1 and primary fibroblasts first evidenced programmed cell death at 40–50μM Resveratrol, IPF fibroblasts did the same only at higher levels >50 μM Resveratrol. These studies showed that both CXCL12 and TGF**β** rescued cells from apoptosis but only at lower concentrations of Resveratrol. These studies also showed that Resveratrol levels ≤50 μM were sub-toxic for both prostate and lung fibroblasts. In addition, the IPF lung fibroblasts tested demonstrated resistance to Resveratrol-induced apoptosis at higher levels (≤100 μM) of Resveratrol than prostate fibroblasts.

### Resveratrol Represses and Reverses TGFβ- and CXCL12-Mediated Prostate Myofibroblast Phenoconversion

**Fig 4:**
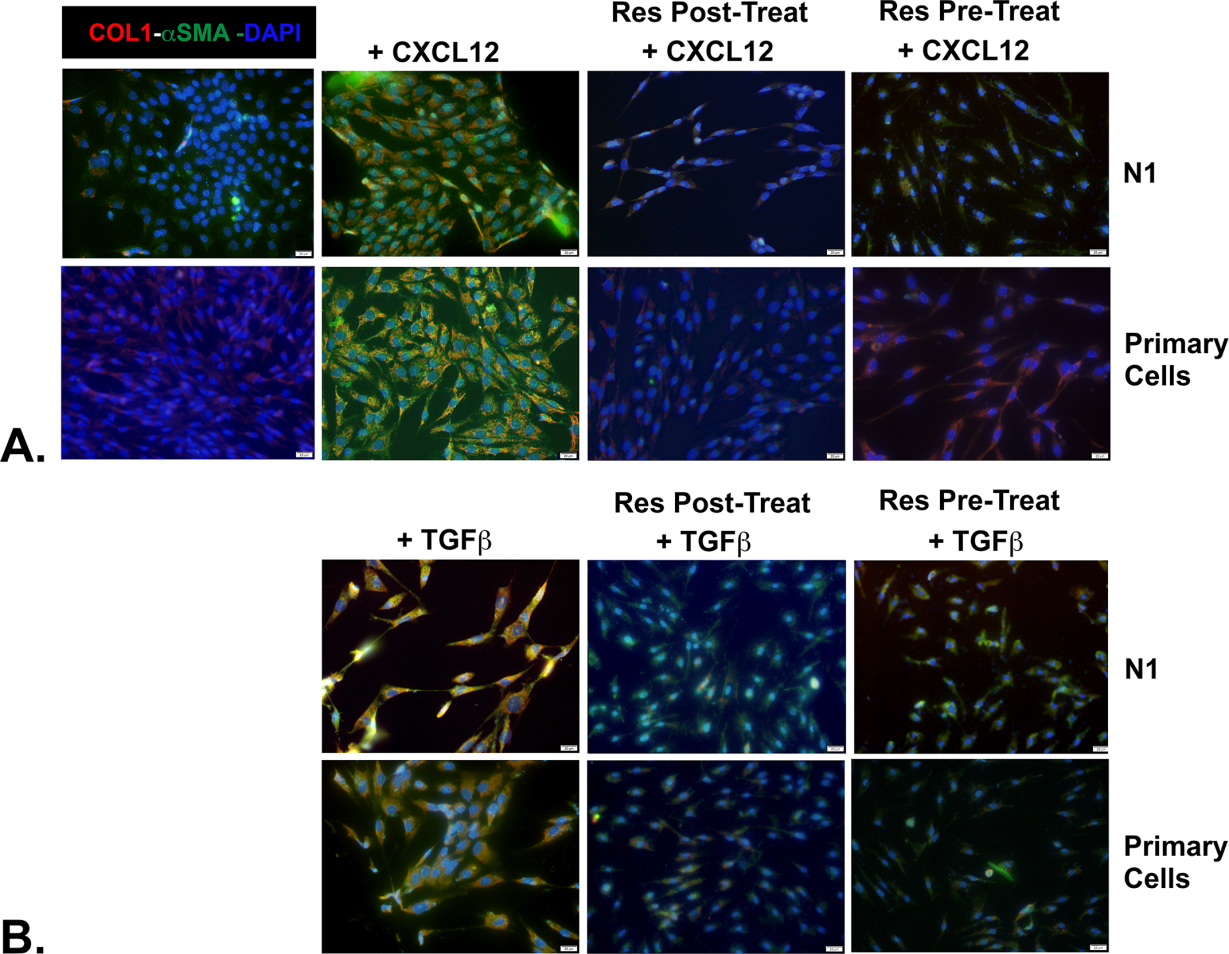
Resveratrol Represses and Reverses CXCL12- and TGFβ-mediated Myofibroblast Phenoconversion of Prostate Fibroblasts. N1 immortalized or primary prostate fibroblasts were seeded, switched to SF media, then treated as indicated below. After fixation, cells were incubated against antibodies to detect COL1 (PE-cy5-conjugated Ab, red) or **α**SMA (fluorescein-conjugated Ab, green), then counterstained with DAPI to image nuclei using immunofluorescence. The images were then merged, and co-expression of COL1 and **α**SMA proteins became evident as orange immunofluorescence. Untreated N1 or primary cells express very low basal levels of COL1 or **α**SMA, whereas cells treated with either CXCL12 or TGF**β** co-express high levels of both proteins. Cells treated with either CXCL12 (**A**) or TGF**β** (**B**) for 24 hr then supplemented (post-treated) with 50 μM Resveratrol for an additional 24 hr demonstrate very little co-expression of COL1 and **α**SMA, though higher levels are observed for TGF**β**-treated cells. Lastly, cells pre-treated with 50 μM Resveratrol for 2 hr followed by supplementation with either CXCL12 (**A**) or TGF**β** (**B**) for 48 hr demonstrate dramatically reduced levels of COL1 and **α**SMA compared to non-Resveratrol treated cells.

**Fig 5:**
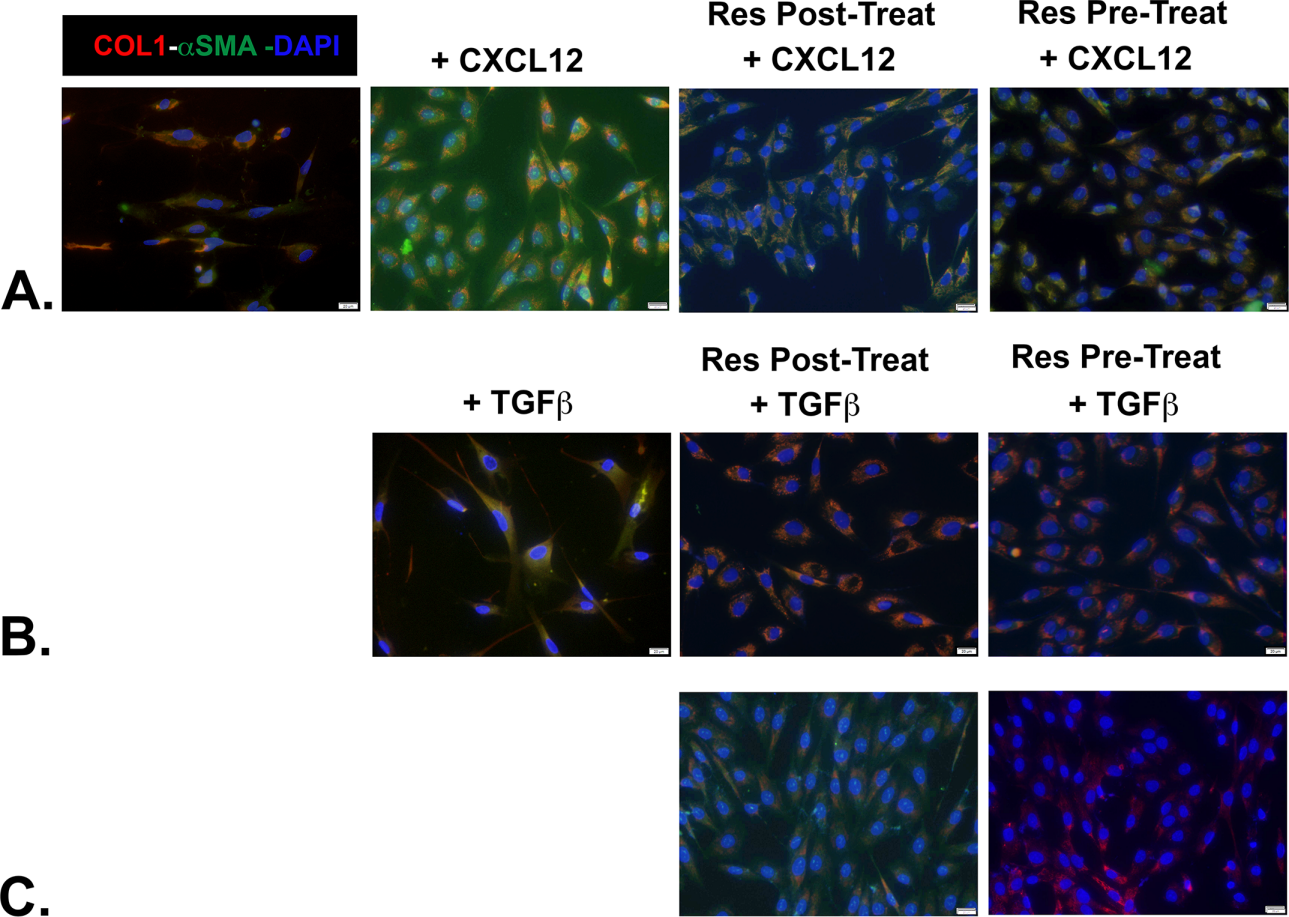
Resveratrol Represses and Reverses CXCL12- and TGFβ-mediated Myofibroblast Phenoconversion of Primary IPF Cells. Primary lung fibroblasts from a patient with clinically significant idiopathic pulmonary fibrosis (IPF) were grown ex vivo. After fixation, cells were incubated against antibodies to detect COL1 (PE-cy5-conjugated Ab, red) or **α**SMA (fluorescein-conjugated Ab, green), then counterstained with DAPI to image nuclei. The images were then merged, and co-expression of COL1 and **α**SMA proteins became evident as orange immunofluorescence. Untreated IPF fibroblasts express very low levels of COL1 or **α**SMA, whereas cells treated with either CXCL12 (**A**) or TGF**β** (**B**) co-express high levels of both proteins. Cells treated with either CXCL12 (**A**) or TGF**β** (**B**) for 24hr then supplemented (post-treated) with 50 μM Resveratrol for an additional 24 hr express reduced but clearly detectable levels of COL1 and **α**SMA. Cells pre-treated with 50 μM Resveratrol for 2 hr followed by supplementation with either CXCL12 or TGF**β** for 48 hr also demonstrate reduced but clearly detectable levels of COL1 and **α**SMA. Finally, cells pre- or post-treated with 100 μM Resveratrol and TGF**β** (**C**) demonstrate nearly ablated levels of COL1 and **α**SMA proteins.

Previous studies had shown that Resveratrol could both repress [25] and revert [26, 27] lung fibrosis. To determine whether Resveratrol could repress or revert prostate myofibroblast phenoconversion, cells were pre- or post-treated with Resveratrol in combination with the pro-fibrotic inducers TGF**β** or CXCL12. In the first set of experiments, N1 immortalized or primary prostate fibroblasts treated with 5ng/ml TGF**β** or 100 pm CXCL12 for 48 hr demonstrated co-expression of COL1 and **α**SMA by immunofluorescence and adoption of a more stellate morphology indicative of myofibroblast phenoconversion (Fig 4). Next, the cells were similarly treated except that sub-toxic levels of 50 μM Resveratrol were added to the culture media after 24 hr. The cells were then assessed for collagen 1 and **α**SMA protein expression after 48 total hr incubation. As seen in Fig 4, both CXCL12- and TGF**β**-treated cells failed to co-express high levels of collagen 1 and **α**SMA and largely retained a fibroblastic spindle-shaped morphology, suggesting that myofibroblast phenoconversion was reversed in these cells though this effect was muted in TGF**β**-treated cells. As expected, Resveratrol also repressed and revereted IPF lung fibroblast to myofibroblast phenoconversion (Fig 5). Thus, as shown for lung fibroblasts, Resveratrol could repress or revert prostate fibroblast to myofibroblast phenoconversion.

### Resveratrol Inhibits the Transcription of Pro-Fibrotic Genes

**Fig 6:**
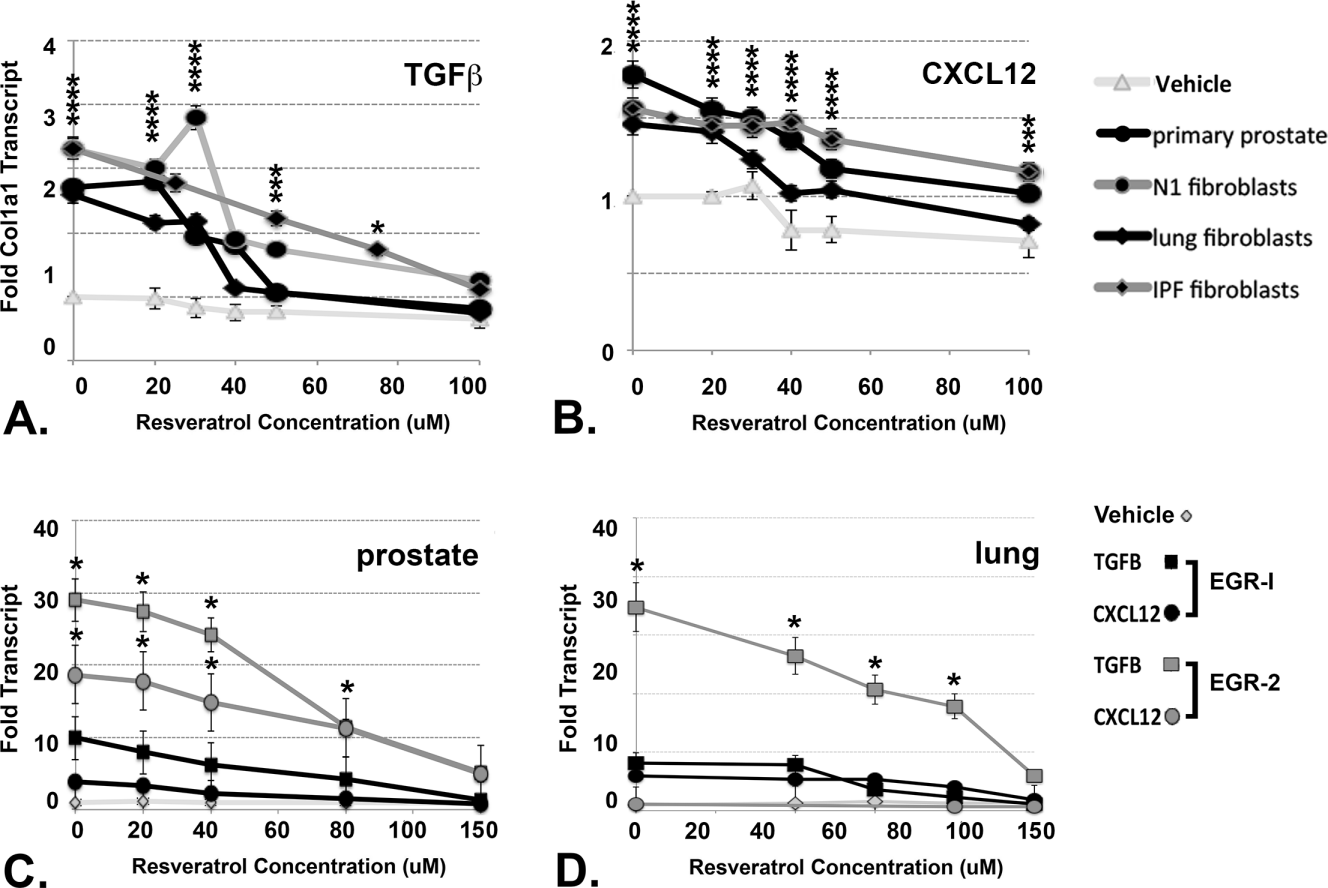
Resveratrol Represses CXCL12- and TGFβ-Mediated *EGR1* Gene Transcription. Primary prostate, N1 immortalized prostate, normal lung, or IPF lung fibroblasts were pre-treated for 2 hrs with ascending doses of Resveratrol, then treated with vehicle, 4ng/ml TGF**β** (A) or 100 pM CXCL12 (B). for 2 hrs. Cells were collected, RNA purified, and subjected to qRT-PCR to detect COL1α1 transcript. Cells treated with CXCL12 or TGF**β** exhibited 1.5–3.0X increased levels of COL1α1 transcript compared to vehicle-treated cells. However, transcript levels decreased significantly with increasing levels of Resveratrol. Similarly treated primary prostate fibroblasts (C) demonstrated increased *EGR1* transcript levels 5X or 10X above that of vehicle-treated cells when treated with CXCL12 or TGF**β**, respectively, and increased *EGR2* transcript levels 20X or 30X above that vehicle-treated cells when treated with CXCL12 or TGF**β**, respectively. Both *EGR1* and *EGR2* transcript levels decreased with increasing Resveratrol concentration. Similarly treated lung fibroblasts (D) exhibited similar responses to CXCL12 or TGF**β** except that CXCL12-treated cells failed to up-regulate *EGR2* transcript levels even in the absence of Resveratrol.

CXCL12 and TGF**β** promote myofibroblast phenoconversion by inducing the transcription of genes that encode proteins such as COL1a1 [14]. Therefore, we studied whether treatment with Resveratrol directly affected the transcription of the *COL1a1* gene in prostate and lung fibroblasts. At concentrations ≥ 50 μM, Resveratrol reduced basal levels of *COL1a1* gene transcription (Fig 6A and 6B). TGF**β** induced COL1a1 gene expression to level 2.5–3.5x higher (Fig 6A), and CXCL12 to levels 1.5–2.0x higher (Fig 6B), than basal levels in all cells tested. This data is consistent with previous data demonstrating that TGF**β** is a stronger pro-fibrotic protein than CXCL12 [14]. However, co-treatment with ≥ 50 μM Resveratrol ablated CXCL12- and TGF**β**-mediated transcription of the *COL1a1* gene (Fig 6A and 6B). Although the correlation between Resveratrol-mediated cellular apoptosis and repression of gene transcription levels cannot be directly compared, it is unlikely that all of the decrease in gene transcription could be attributed purely to cellular apoptosis. This is because the overall effects of Resveratrol treatment are quite discrepant, accounting for only a 5–20% increase in cellular apoptotic rate compared to 0.5–2.5x reduction in *COL1a1* gene transcription.

The *COL1a1* [28]and *COL1a2* [29] gene promoters are complex and harbor multiple potential transcription factor binding sites. Among these are the Egr-1 and Egr-2 proteins have been shown to have been shown to promote fibrosis through binding to consensus sequences in the gene promoter, and induce the transcription, of the COL1a1 and COL1a2 genes [30, 31]. Our laboratory has previously shown that activation of the CXCL12/CXCR4 axis promotes MEK/ERK signaling, Elk-1 phosphorylation, and consequent *EGR1* gene transcription in prostate epithelial cells [32]. Resveratrol's anti-fibrotic properties have, in part, been attributed to its inhibitory effect on *EGR1* gene transcription [33, 34]. Therefore we examined whether Resveratrol affected *EGR1 and/or EGR2* gene transcription in primary prostate and lung fibroblasts. These studies showed that primary prostate and lung cells treated with 100 pM CXCL12 demonstrated elevation of *EGR1* transcript levels by 4-6X or with 4ng/ml TGF**β** by 7-10X over basal levels (Fig 6C). *EGR1* gene transcript levels progressively diminshed upon co-treatment with increasing doses of Resveratrol. *EGR2* gene transcription was induced by treatment with TGF**β** to levels 30X higher than basal levels in primary prostate and lung cells and diminshed upon co-treatment with increasing doses of Resveratrol (Fig 6C). In primary lung cells, however, EGR2 transcription was stimulated by treatment with TGF**β** but not CXCL12. This finding suggests the potential role(s) of Egr-1 and Egr-2 proteins in collagen gene transcription may differ between prostate and lung fibroblasts.

## Discussion

Fibrotic changes in tissue architecture contribute to dysfunction and disease in multiple organ systems, including pancreatic dysfunction in type 2 diabetes [4, 5], chronic obstructive pulmonary disease [6, 7], cirrhotic nonalcoholic fatty acid liver disease [8, 9], and Crohn's disease, which is part of the spectrum disorder termed inflammatory bowel disease [10, 11]. Our group recently showed that peri-urethral prostatic fibrosis is associated with lower urinary symptoms and urinary voiding dysfunction in mice and men [13, 35]. Based on the multiple organ systems that can be adversely affected by fibrosis, there is a clear need for the development and testing of anti-fibrotic therapies that can target abnormal myofibroblast accumulation and ECM deposition.

A recent review by Wynn and Ramalingam describes multiple anti-fibrotics in pre-clinical development or human clinical trial [36]. Many of these small molecules and biologics target TGF**β** activity, though others target the ECM, intracellular enzymes, inflammatory mediators, and oxidative stress. Resveratrol appears to affect some of these same targets, including inflammation and oxidative stress [37, 38]. Recent studies have shown that resveratrol acts to suppress collagen synthesis and ameliorate fibrosis specifically through suppressing TGF**β/**Smad activity [39–42] and/or MEK/Erk signaling [25, 43], which has been shown to induce transcription of the *COL1a2* gene promoter [44]. Studies accomplished *in vivo* show that Resveratrol can blunt or revert chemically-induced lung fibrosis in rodent models [27, 42, 45]. Based on these studies, the current investigation intended to determine whether prostate fibroblasts would respond to Resveratrol treatment in a manner similar to that already demonstrated for lung fibroblasts, e.g., repress or revert myofibroblast phenoconversion in vitro. This study also sought to determine whether these effects could be achieved with low or no toxicity to prostate fibroblastic cells, hence, whether Resveratrol might have therapeutic value to treat lower urinary tract fibrosis as a contributing pathobiology to urinary voiding dysfunction.

The results of these studies showed that doses of Resveratrol in the range of 40–50 μM were well-tolerated by N1 immortalized and primary lung and prostate fibroblasts. These low concentrations were sufficient to reduce TGF**β-** and CXCL12-mediated cellular proliferation, increase the basal cellular apoptotic rate, and repress *COL1* gene transcription or COL1 or **α**SMA protein expression. Moreover, these low concentrations were sufficient for repression or reversion of myofibroblast phenoconversion. Higher levels of Resveratrol (≥100 μM) were required to produce the same effects in IPF lung fibroblasts. These data suggest that low levels of Resveratrol might have some efficacy for the treatment of prostate fibroblast phenoconversion to myofibroblasts.

N1 immortalized and primary prostate fibroblasts as well as IPF fibroblasts demonstrated similar proliferative responses to treatment with CXCL12 and TGF**β**, which were inhibited with increasing doses of Resveratrol. Similarly, increasing doses of Resveratrol induced apoptosis for the same 3 cultured cell populations, which was repressed by treatment with CXCL12 or TGF**β**. These studies suggest that prostate and lung fibroblasts respond similarly to Resveratrol, implying that findings relevant to lung fibrosis repression or reversion by Resverarol may apply to prostatic fibrosis as well.

Resveratrol has been tested in human clinical trials to determine efficacy for treating colon carcinoma, type 2 diabetes, cardiovascular and coronary artery disease, and other conditions [46]. Although the dosages and modes of ingestion of Resveratrol varied markedly between these trials, a common them was a measureable reduction in markers of oxidative stress and inflammation [46]. Perhaps more pertinent to the present study, a double-blind controlled trial randomized 50 patients with Nonalcoholic fatty liver disease (NAFLD) into 2 groups: those that received placebo, and those that received 500mg Resveratrol for 12 weeks. In this study, Resveratrol supplementation was associated with a significant reduction in liver enzyme alanine aminotransferase, inflammatory cytokines, nuclear factor κB activity, serum cytokeratin-18, and hepatic steatosis grade, as compared with placebo supplementation (P < .05) [47]. This is an encouraging finding, as NAFLD often precedes and is considered causative to hepatic fibrosis and cirrhosis [8, 9]. Combined with its relative low toxicity [46, 48], the NAFLD data suggests a potential for the administration of Resveratrol as a means to prevent or treat extant human organ fibrosis.

Egr-1 is a pro-fibrotic protein that can promote the expression of the COL1a2 gene which, together with the COL1a1 gene, encodes the triple-stranded collagen 1 protein secreted by myofibroblasts during fibrogenesis [49, 50]. The study reported here shows that Resveratrol can repress *EGR1* gene transcription in CXCL12- or TGF**β**-treated primary prostate or lung fibroblasts. However, *EGR2* gene transcription was not repressed by Resveratrol in TGF**β**-treated lung fibroblasts. This suggests that the role(s) of Egr-1 and Egr-2 may differ between prostate and lung myofibroblast phenoconversion.

It is clear from the literature that the effects of Resveratrol on *EGR1* gene transcription are concentration dependent [51], with some reports stating that lower (≤20 μM) concentrations of Resveratrol increase, while higher (≥50 μM) concentrations decrease, *EGR1* gene expression [33, 52–54]. Therefore, it is possible that the present study did not observe Resveratrol-mediated *EGR1* gene induction due to the higher concentration of Resveratrol used compared to other studies. Moreover, the *EGR1* gene promoter is under complex regulation [44, 55] and there is evidence that whether Egr1 plays a pro- or anti-fibrotic role can be context dependent [30]. Therefore, multiple factors likely play into whether EGR1 gene repression comprises an aspect of Resveratrol-mediated inhibition or reversion of myofibroblast phenoconversion.

Limiting factors in the present study include the testing of 2 out of many pro-fibrotic proteins; the investigation of myofibroblast phenoconversion in fibroblasts derived from only 2 organs, lung and prostate; and the limitation of these studies to cells *in vitro*. Even so, important information regarding the efficacy of Resveratrol to not only repress but actually revert the myofibroblast phenoconversion of prostate fibroblasts has been obtained. Taken together, these data suggest that, like lung fibroblasts, prostate fibroblast to myofibroblast phenoconversion can both be repressed and reversed by Resveratrol treatment. Thus, anti-fibrotic therapeutics might be efficacious for the treatment of LUTD.
